# Supplementary material for: Evolutionary Fate of the Opine Synthesis Genes in the Arachis L. Genomes
Source: Biology (Basel). 2024 Aug 9;13(8):601. doi: 10.3390/biology13080601 (PMC11351324; doi:10.3390/biology13080601)
Supplement: Supplementary file 1 [file biology-13-00601-s001.zip › Table S2.pdf]

Sequences of primers

Sequence of primer	Aim of the experiment
Ad_cusF GGGGGTTGCTACTAGAGTAT<br>Ad_cusR TCTGCCCCACAAATACCAT	Coding sequence study
Ad_cus_reR GCTAGCTCCGCCACCACATT<br>Ad_cus_reF GGTTGCACCGTTGGTGTGG	Real-time RT-PCR
gapdhF GGTGCCAAGAAGGTTGTGAT<br>gapdhR CAAGGCAGTTGGTAGTGCAA	Reference for Real-time RT-PCR
